# An unusual presentation of anetoderma: a case report

**DOI:** 10.1186/1471-5945-4-9

**Published:** 2004-08-19

**Authors:** Shahin Aghaei, Manouchehr Sodaifi, Fatemeh Sari Aslani, Nazila Mazharinia

**Affiliations:** 1 Department of Dermatology, Shiraz University of Medical Sciences, Shiraz, Iran; 2 Department of Pathology, Shiraz University of Medical Sciences, Shiraz, Iran; 3 Burn Hospital Shiraz University of Medical Sciences, Shiraz, Iran

**Keywords:** Anetoderma, Extremity, Acra, Cryotherapy

## Abstract

**Background:**

Anetoderma is a benign condition with focal loss of dermal elastic tissue resulting in localized areas of flaccid or herniated saclike skin. Currently, anetoderma is classified as either primary (idiopathic), or secondary anetoderma (which is associated with a variety of skin conditions, penicillamine use, or neonatal prematurity). Lesions appear on the upper arms, trunk, and thighs.

**Case presentation:**

We report a 14-year-old boy, which was noticed to have had multiple, white, non-pruritic areas on the acral sites of upper and lower extremities for two years. In physical examination, the patient had normal mental development. Skin lesions consisted of scattered, white to skin-colored papules, less than 1 cm in diameter, and with central protrusion, with distribution on dorsal part of the index finger, forearms, distal portion of thighs and calves. Lesions were detected neither on the trunk nor the proximal areas of extremities. There are no sensory changes associated with the lesions. Otherwise, his general health was good. He did not have any medication consumption history. Family history was negative. Laboratory examinations were within normal limits. Skin biopsy from one of his lesions was done, that confirmed the diagnosis of anetoderma.

**Conclusions:**

In summary, we report a case of anetoderma on unusual sites of the skin. We could not find similar reports of anetoderma developing on distal extremities without involvement of the upper trunk and proximal arms, in the medical literature.

## Background

The term anetoderma (anetos = slack) refers to a circumscribed area of slack skin associated with a loss of dermal substance on palpation and a loss of elastic tissue on histological examination [1].

In the past, cases of primary anetoderma were divided into the Jadassohn-Pellizari type, in which the lesions are preceded by erythema or urticaria, and the Schweninger-Buzzi type, in which there are no preceding inflammatory lesions. This is now of historical interest only, because in the same patient some lesions may be preceded by inflammation and the others may not, and the prognosis and histology are identical in the two types [2,3].

Anetoderma is a rare disorder that in the most usual form develop on the trunk, thighs and upper arms, less commonly on the neck and face and rarely elsewhere.

The scalp, palms and soles are usually spared. We report a patient with anetoderma whose lesions present on distal extremities consisting of hands and calves.

## Case presentation

A 14-year-old boy was noticed to have had multiple white, non pruritic area on his distal extremities for two years. The lesions consisted of whitish papules and depressed areas with central protrusion.

**Figure 1:**
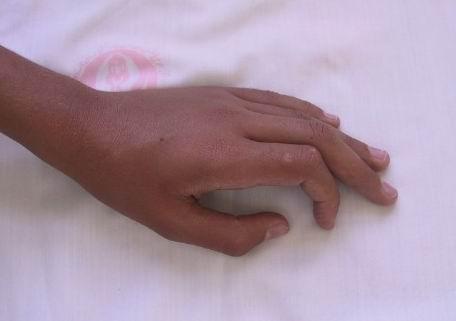
Solitary lesion over dorsum of left index finger.

**Figure 2:**
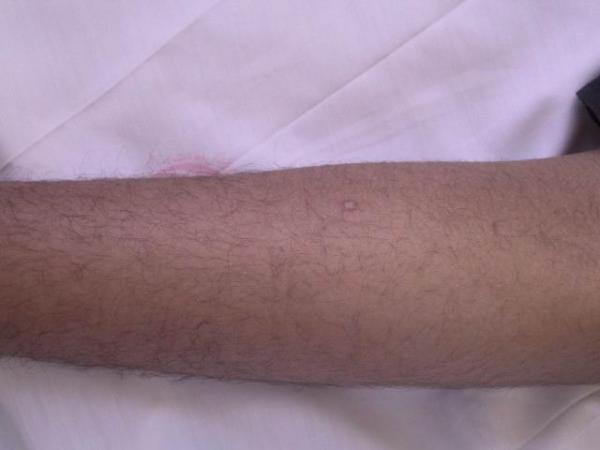
An anetodermic lesion on forearm.

**Figure 3:**
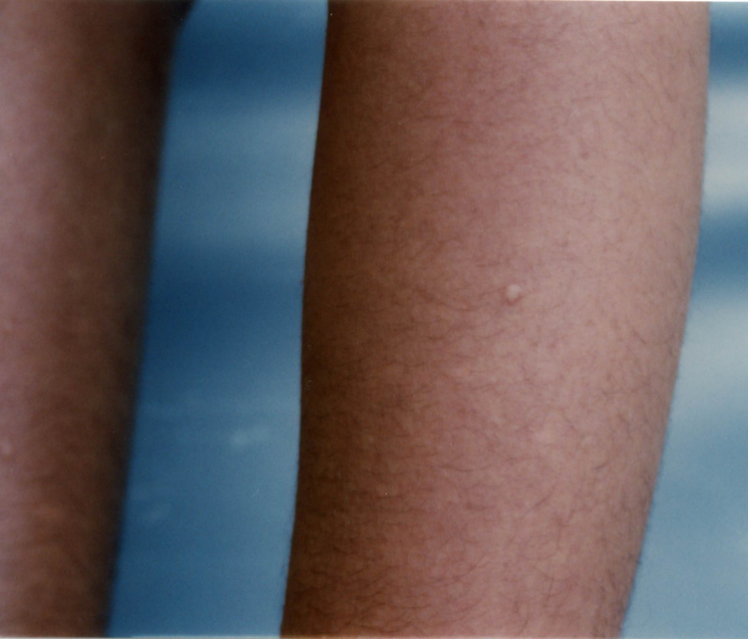
An anetodermic lesion on lateral aspect of left lower leg (before cryotherapy)

On clinical examination, the otherwise healthy looking patient's general appearance and mental state had lumbar lordosis, laxity in large joints, tibia vara, high-arched palate, and dental misalignment. Skin lesions consisted of scattered, white to skin-colored papules, less than 1 centimeter in diameter, and with central protrusion, that were distributed on the dorsum of fingers (Fig 1), forearms (Fig 2), distal portion of the thighs and on the calves (Fig 3). No lesions on the trunk or proximal areas of extremities were detected. Palms, soles, dorsum of feet and mucosal membranes were spared. No sensory changes associated with the lesions. He did not have any history of medication consumption. Family history was negative.

**Figure 4:**
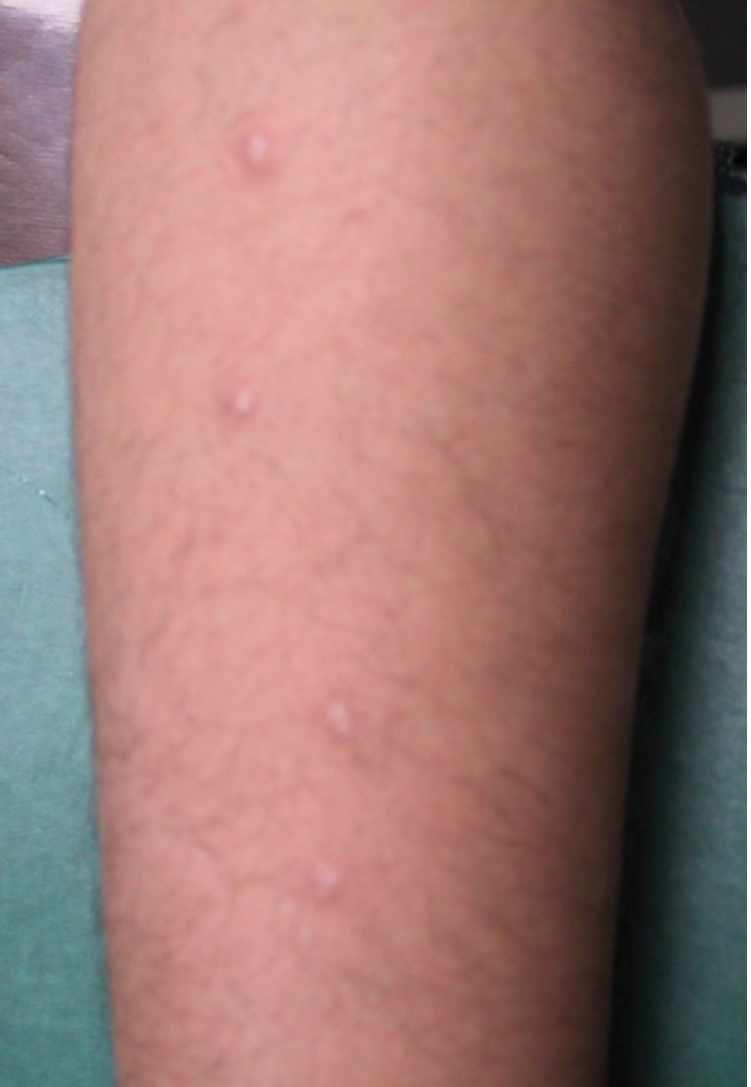
Anetoderma lesions some minute after cryotherapy

**Figure 5:**
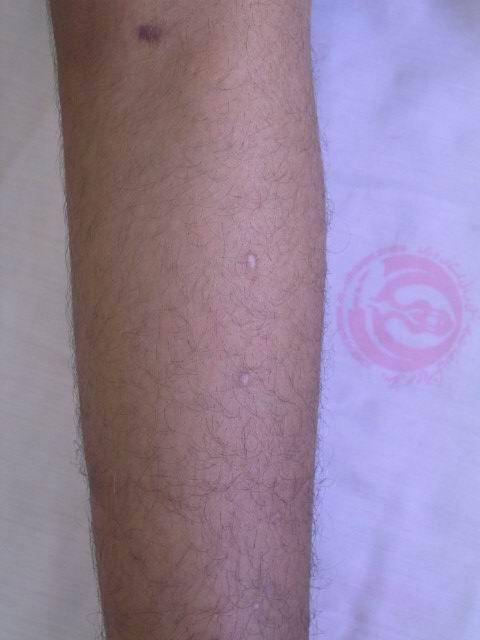
The same view as Fig. 4 after several sessions of cryotherapy (the 2 lower lesions near completely been resolved).

**Figure 6:**
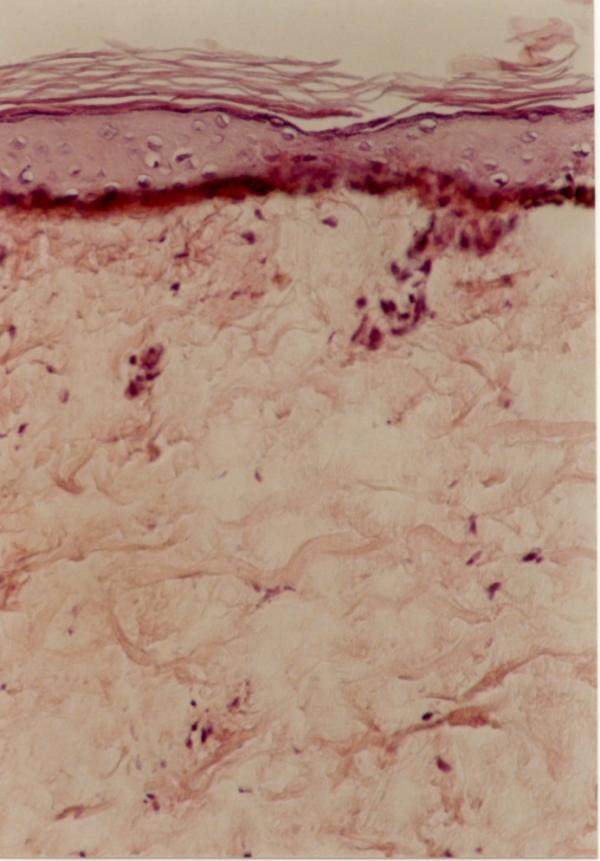
Faintly eosinophilic separated collagen bundles in upper dermis (H&E ×250).

**Figure 7:**
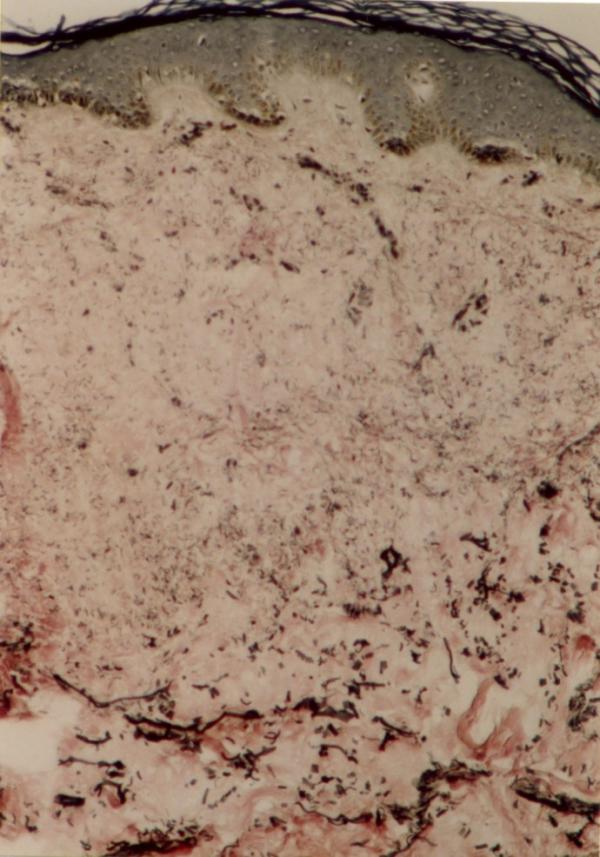
Severely decreased elastic fibres in superficial and mid dermis (Verhoeff-van Gieson stain ×250).

**Figure 8:**
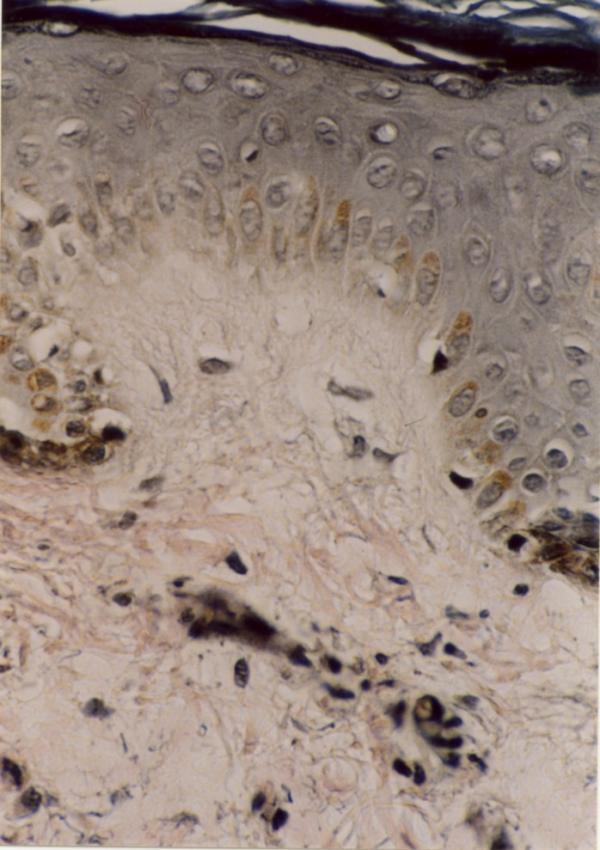
Severely decreased elastic fibres in superficial dermis (Verhoeff-van Gieson stain ×400).

Laboratory examinations consisting of complete blood count, urinalysis, and blood chemistry including erythrocyte sedimentation rate and liver function tests were within normal limits. Antinuclear antibody was negative. The patient had no risk factor for AIDS or syphilis, so we did not request HIV or VDRL test. Hepatitis B surface antigens were not detected. Immunological assays consisting of IgM, IgA, IgG, IgE, C3, and C4 levels were normal. An induration of 0.5 centimeter in diameter was observed after tuberculin testing. Chest x-ray film was normal. The skin biopsy was done. Haematoxylin and eosin stained section showed faintly eosinophilic separated collagen fibers in the upper and mid-dermis (Fig 6). Verhoeff-vanGieson stained sections showed a marked decrease or in some areas total absence of elastic fibres, in both superficial and mid-dermis. Elastic fibres around the vessels in the affected areas are also fragmented and markedly decreased (Fig 7,8). We tried to treat the patient with liquid nitrogen cryotherapy by means of cotton-tip applicator for two freeze-thaw cycles (freeze time, 10–15 seconds), in 6–8 sessions weekly; which obtained moderate improvement in some early-onset lesions with no frank atrophy (Fig 4 and 5).

## Discussion

Anetoderma, which was first described by Jadassohn in 1892, is characterized by localized areas of loss of substance and elastic tissue with flaccid skin and often leads to a herniation phenomenon [2]. We could not find similar reports (other than anetoderma-like changes on distal extremities secondary to hamartomatous congenital melanocytic naevi) [4] of anetoderma developing on distal extremities without involvement of the upper trunk and proximal arms, in the medical literature.

This rare disorder occurs mainly in women aged 20–40 years, but is occasionally reported in younger and older patients of both sexes. It is perhaps more frequent in central Europe than elsewhere, which suggests a possible relationship to chronic atrophic acrodermatitis (due to *Borrelia *species) in some cases. In the most usual form, crops of round or oval, pink macules 0.5–1 centimeter in diameter develop on trunk, thighs and upper arms, less commonly on the neck and face and rarely elsewhere[3]. The scalp, palms and soles are usually spared. Each macule extends for a week or two to reach the size of 2–3 centimeter [3]. Sometimes there are larger plaques of erythema, and nodules have also been reported as primary lesion [5]. The number of lesions varies widely, from less than five to one hundred or more [3]. The lesions remain unchanged throughout life, and new lesions often continue to develop for many years. If the lesions coalesce, they form large atrophic areas, which are indistinguishable from acquired cutis laxa [3]. They may become confluent, to cover large areas, especially at the roots of the limbs and on the neck [3].

Although infrequently reported, anetoderma may occur in families, and patient must be examined for associated systemic abnormalities for thorough assessment of their skin disorders. In familial anetoderma, there were associated ocular, gastrointestinal or orthopedic anomalies in the affected patients or in any other family members, but causes without them have been reported [8]. Although isolated and perhaps coincidental, these abnormalities could be related to the same process that produces the lesions of anetoderma [3].

Primary anetoderma can be inherited, but it has also been described in association with prematurity, lupus erythematosus, antiphospholipid syndrome, and with decreased serum levels of alpha-1-antitrypsin [6,7,9,10] and [11]. Secondary anetoderma develops over other dermatoses are shown in Table 1.

**Table 1:** Dermatoses Associated with Anetoderma

Syphilis [3]	Sarcoidosis [17]	Granuloma annulare [25]
Tuberculosis [3]	Acne vulgaris [17]	Hepatitis B virus immunization [26]
Xanthomas [3]	Leprosy [17]	Primary Sjogren's syndrome [27]
Nodular amyloidosis [3]	Lupus erythematosus [17, 21]	Lichen planus [28]
Melanocytic naevi [4]	Pilomatricoma [18, 19]	Insect bites [28]
Low serum level of α-1-antitrypsin [6]	Prurigo nodularis [20]	Lupus profundus [30, 31]
Antiphospholipid syndrome [6, 11, 1nd 24]	Cutaneous plasmacytoma [21]	Discoid lupus (with herediyary C2 defficiency) [32]
Recurrent deep vein thrombosis [7]	Benign cutaneous lymphoid hyperplasia [21]	Pityriasis versicolor [33]
History of Graves' disease [7]	Urticaria pigmentosa [22, 23]	Dermatofibroma [34]
Familial type [8]	Perifolliculitis [23]	Penicillamine-induced [34, 40]
Prematurity [10]	Varicella [24]	HIV-infection [42]

The differential diagnosis of anetoderma includes other focal dermal atrophies and miscellaneous diseases that must be differentiated from the skin herniation phenomenon of anetoderma [12], are shown in Table 2.

**Table 2:** Differential Diagnosis of Anetoderma [12-16]

Atrophic scars	Discoid lupus erythematosus
Lichen sclerosus et atrophicus	Atrophoderma of Pasini and Pierini
Corticosteroid-induced atrophy	Perifollicular macular atrophy
Morphea	Perifollicular atrophoderma
Atrophoderma vermiculare	Striae distensae
Focal dermal hypoplasia	Naevus lipomatosus
Connective tissue naevus	Neurofibromas
Cutis laxa	postinflammatory elastolysis
mid-dermal elastolysis	Granulomatous slack skin
acrodermatitis chronica atrophicans	

Atrophoderma of Pasini and Pierini is a major source of confusion both etymologically and clinically. Patients have larger lesions with a sharp peripheral border dropping into a depression with no outpouching. On biopsy, elastin is normal, while collagen may be thickened, but this finding is difficult to quantify [12]. Perifollicular atrophoderma is most prominent on the dorsa of the hands and often is associated with multiple basal cell carcinomas and hair abnormalities in the Bazex syndrome [13]. Perifollicular atrophy also has been described in extreme forms of keratosis pilaris, in which large keratin plugs may produce a dilated patulous follicle. This condition usually found on the cheeks of young children. Both of these lesions mimic perifollicular anetoderma but lack elastin changes [12]. In focal dermal hypoplasia thinning or absence of dermis, rather than changes in elastin fibres, accounts for the proximity of the subcutis to the epidermis [12]. Cutis laxa, postinflammatory elastolysis [14], and mid-dermal elastolysis [15] share with anetoderma the property of cryptogenic loss of elastic fibres.

Elastase-producing strains of staphylococcus epidermidis have been held responsible for perifollicular macular atrophy. Anetoderma has also been reported in 5 patients with false-positive syphilis serology, 3 of who also fulfilled the criteria for the antiphospholipid syndrome [29]. Its pathogenesis is not yet clearly established, but immunological mechanisms could play an important role in dermal elastolysis [35]. The association of primary anetoderma with decreased levels of alpha-1-antitrypsin may be of significance: Alpha-1-antitrypsin inhibits neutrophil elastase and its reduction may cause increased elastic activity and elastin breakdown. Phagocytosis of elastic fibres by macrophages has been found in primary anetoderma [36]. No antibodies have been demonstrated against elastic fibres [37].

Venencie et al. [38], suggested that the degradation of elastic fibres in patients with anetoderma is caused by enhanced expression of progelatinases A and B and production of the activated form of gelatinase A, and that the lack of control of these enzymes by tissue inhibitors of metalloproteinases is probably a key factor in the development and duration of anetodermic lesions.

Ghomrasseni et al. [39], demonstrated that for the five samples of anetodermic skin, matrix metalloproteinase-1 (MMP-1) levels were significantly higher compared with the uninvolver cultures and the healthy samples. A significant increase of tissue inhibitors of metalloproteinase (TIMP-1) expression was also observed in the affected cultures of explants. The study demonstrated a significant increase in the production of gelatinase A (MMP-2), and no significant production of TIMP-2 in lesional skin compared with the samples from the two healthy donors.

Penicillamine-induced anetoderma has also been reported [3,34] and [40]. Penicillin and the antifibrinolytic drug ε-aminocaproic acid have been advocated [41], but Venencie et al. [3] studied 16 patients and found that no treatment was beneficial once the atrophy had developed. However, the wrinkled skin appearance in our patient had been present for 2 years, and his lesions did not show any signs of inflammation or pre-existing conditions like melanocytic naevi.

## Conclusions

In summary, we report a case of anetoderma with lesions on unusual sites. We did not find similar reports of acral anetoderma in the medical literature.

According to this paper liquid nitrogen cryotherapy has moderate efficacy in the treatment of some of the early anetoderma lesions, without frank atrophy.

## Competing interest

None declared.

## Pre-publication history

The pre-publication history for this paper can be accessed here:


